# Implementation and performance analysis of QPSK system using pocket double gate asymmetric JLTFET for satellite communications

**DOI:** 10.1038/s41598-023-29864-7

**Published:** 2023-02-21

**Authors:** Lokesh Boggarapu, Lakshmi B

**Affiliations:** 1 School of Electronics Engineering, Vellore Institute of Technology, Chennai, 600127 India; 2 Centre for Nano Electronics and VLSI Design and School of Electronics Engineering, Vellore Institute of Technology, Chennai, 600127 India

**Keywords:** Nanoscale devices, Nanoscale materials

## Abstract

This work is intended to design a quadrature phase shift keying (QPSK) system starting from the device design, characterization and optimization which is then followed by the circuit level implementation and finally the system level configuration. Tunnel Field Effect Transistor (TFET) technology came into existence because of the inability of CMOS (Complementary Metal Oxide Semiconductor) to produce reduced leakage current (I_off_) in the subthreshold regime. With the effects of scaling and requirement of high doping concentrations, TFET is not capable to produce stable reduction in I_off_ due to the variation in ON and OFF current. To improve the switching ratio of the current and to obtain good subthreshold swing (SS) by overcoming the limitations of junction TFET, a new device design is proposed for the first time in this work. A pocket double gate asymmetric Junction less TFET (poc-DG-AJLTFET) structure has been proposed in which uniform doping is used to eliminate the junctions and a pocket of length 2 nm made of Silicon–Germanium (SiGe) material has been introduced to improve the designed structure performance in the weak inversion region and increase the drive current (I_ON_). The work function has been tuned to produce the best results for poc-DG-AJLTFET and with our proposed poc-DG-AJLTFET, effects of interface traps are eliminated as against conventional JLTFET structures. The notion that low-threshold voltage device yields high I_OFF_ has been proved wrong with our poc-DG-AJLTFET design, as it produced low threshold voltage with lower I_OFF_ which reduced the power dissipation. Numerical results show that drain induced barrier lowering (DIBL) of 2.75 mV/V is achieved which could be less than 35 times required for short channel effects to be minimum. In terms of gate to drain capacitance (C_gd_), it is found that ~ 10^3^ reduction which greatly improves device inertia to internal electrical interference. Also, improvement in transconductance is achieved by 10^4^ times, 10^3^ times improvement in I_ON_/I_OFF_ ratio, and 400 times higher unity gain cutoff-frequency (f_t_) which would be required by all communication systems. The Verilog models of the designed device are used to construct the leaf cells of quadrature phase shift keying (QPSK) system and the implemented QPSK system is taken as a key evaluator in the performance evaluation in terms of propagation delay and power consumption of poc-DG-AJLTFET in modern satellite communication systems.

## Introduction

As technology node scales down, it fuels various engineering techniques to overcome the short channel effects (SCEs) and gain better control of the channel to increase the drive current (I_ON_) and reduce the leakage current (I_OFF_) in the device. Unfortunately, conventional metal oxide semiconductor field effect transistors (MOSFETs) are not able to overcome SCEs and produce a stable and high current switching ratio (I_ON_/I_OFF_), degraded SS, high DIBL, and highly sensitive electrical characteristics. For this, similar device structures such as Silicon on Insulator MOSFETs (SOI MOSFETs) for low SS and low leakage currents with less threshold voltages being applicable provided that the gate lengths are long^1–4^. But, Semiconductor device engineers are continuously investigating new devices and configurations to reduce SCEs and thus improve device performance for short channel devices. In one such investigation, Tunnel field effect transistors (TFETs) came into existence which can produce high switching ratio and steeper subthreshold swing (SS)^5–7^. However, Silicon (Si) TFETs with SiO_2_ as gate oxide, offer poor electrostatics and experimental results show that the on-state currents in TFETs are typically lower than that of MOSFETs^8–11^. So, with low on-state current as drawback, new configurations, and materials for TFET are proposed, heterojunction tunnel field effect transistor (HTFET), L and U channel TFETs (LTFET & UTFET) and usage of III-V group materials^12,13^ for tunneling devices. For these new tunneling devices, high doping concentrations are necessary to increase tunneling probability in order to produce high I_ON_, but with high doping concentrations in source/drain regions where p-n junctions have significant variability issues, leading to fabrication challenges. To avoid these problems and to ease fabrication, new technology of TFETs has been proposed which are named as Junction less Tunnel field effect transistors (JLTFETs)^14–16^. Since these use uniform doping concentrations, the p-n junctions are eliminated and thus show less variability in the drive and leakage currents^17,18^.

Typical structure of JLTFET with n^+^ source/drain and n^-^ channel are converted into p^+^-i-n^+^ regions with polarity gate and control gate with different work-functions as driving contacts for the device^19,20^. But it has been observed in many of the JLTFET devices that though tunneling probability and channel control is high, introduction of polarity gate over source region can cause adverse change in electrical characteristics when interface traps are introduced into the device^21,22^. This effect can be avoided by completely removing the so-called polarity gate. For this, we proposed a device called pocket double gate asymmetric Junction less TFET (poc-DG-AJLTFET) of n^+^-p^+^-n^+^ regions with pocket gate (PG) and control gate (CG). The consequences of removing polarity gate and overcoming of those consequences are discussed further in Section "Device description and simulation". Also, to improve the electrical characteristics, a pocket would be introduced in the device design^23^. For the device to perform better in analog circuits, various parameters such as output resistance (R_out_), trans-conductance (g_m_), unity gain cutoff-frequency (f_t_) play a significant role^24^. Moreover, to obtain good performance in terms of analog parameters, Gallium Nitride (GaN) has been chosen as material for the whole device^25^. Recently, Sharma et al proposed a DGJLTFET with a new approach for wireless communication systems showing promising I_ON_ & high switching ratio^26^.

The device in this work is designed in such a way that it can be suitable for analog applications without compromising on the DC parameters such as SS & I_ON_/I_OFF_ ratio. For the first time, a poc-DG-AJLTFET has been designed to achieve optimal SS and a high I_ON_/I_OFF_ ratio, high (g_m_) to I_ON_ ratio, very low I_OFF_ to make the analog suited structure also suitable for digital systems such as quadrature phase shift keying (QPSK) communication system. Both analog and digital parameters are carefully analyzed to make the device suitable for mixed signal applications. In circuit level, digital part of QPSK system has been implemented. The corresponding leaf cells NAND, X-OR gates and Delay flip-flops (DFFs) have been designed and simulated considering the worst case so as to produce best results in all scenarios. The designed leaf cells (NAND, X-OR gates) have been modified to consume low power using pass transistor logic. The fully designed QPSK system is then simulated and the parameters, propagation delay and power consumption have been measured as the key evaluators of the designed poc-DG-AJLTFET.

The framework of the paper is as follows: section "Device description and simulation" provides the device structure and its corresponding parameter spacing. Section "Results and discussion" provides results & discussion. Section "Performance analysis of QPSK system" provides performance analysis of QPSK system. Section "Conclusion" gives the conclusion.

## Device description and simulation

**Figure 1:**
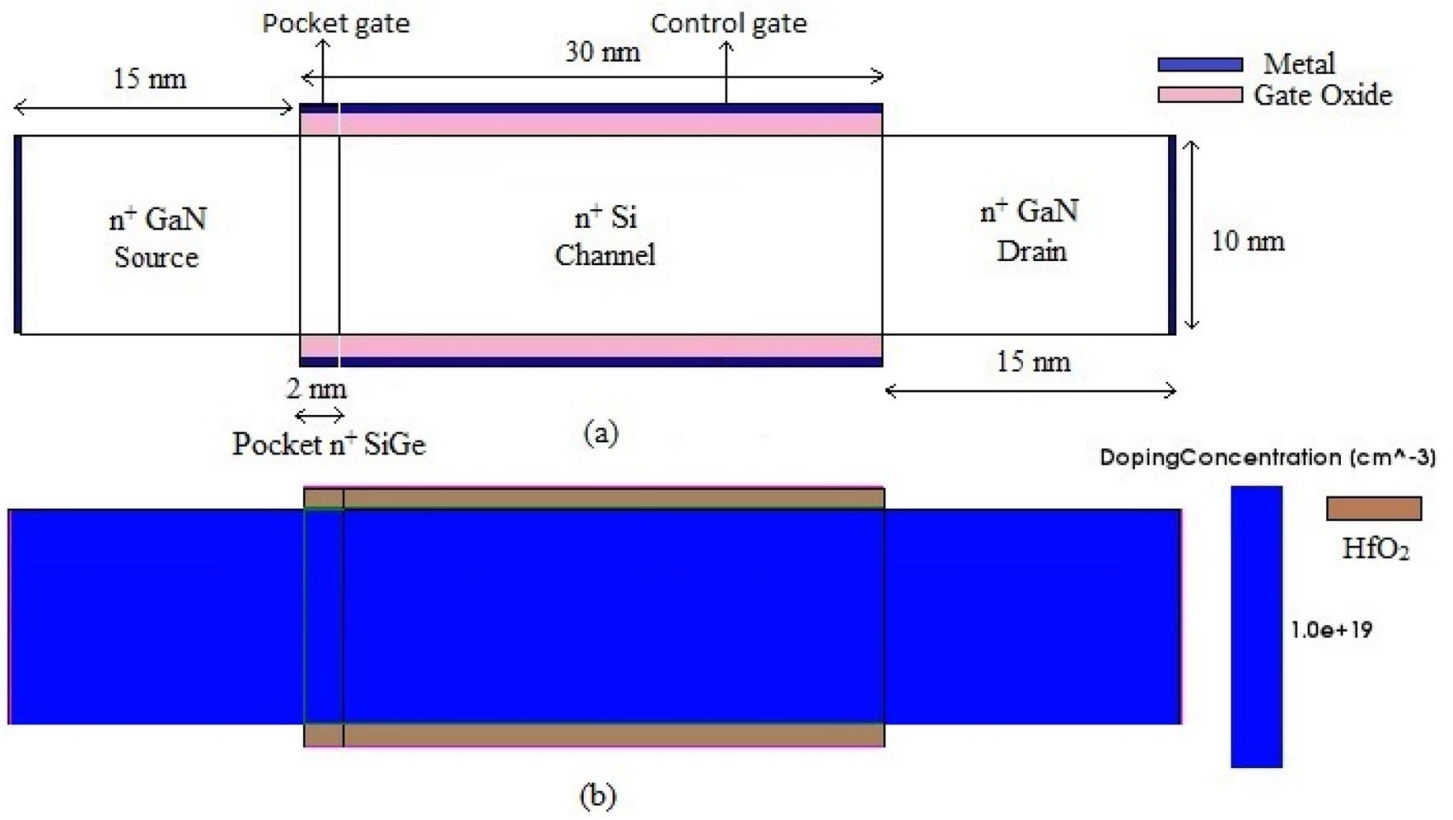
(**a**) Schematic structure of poc-DG-AJLTFET. (**b**) Simulated structure of poc-DG-AJLTFET.

In this work, 2-D Sentaurus TCAD has been used to design and simulate poc-DG-AJLTFET^27^. Figure 1a gives the schematic of the device; Fig. 1b shows the simulated structure of poc-DG-AJLTFET which has been proposed for the first time and is calibrated against the published results^28,29^. The device is made of GaN except for the channel and pocket which are made of Silicon and Silicon Germanium (SiGe) respectively and is doped uniformly with a doping concentration of 1 × 10^19^ cm^−3^. The main reason for choosing GaN for source & drain in Junction less TFETs is that it would produce better electrical characteristics as that of conventional Si and Ge^30^. But, by choosing GaN as body material for a device with Si or Ge as interface to GaN, several issues occur in the stages of fabrication. It is known that GaN is a very hard wurtzite material with knoop hardness of 14.21 gigapascal (GPa) and it has a high dislocation density in the order of 10^8^ to 10^10^ defects per square centimeter when grown on Si, while Si has a diamond cubic crystal structure^31^. Though there is a mismatch between them, GaN can be grown on Si but the latter would change the former growth in such a way that it would become brittle. To avoid this, a 2-step process of developing buffer layer of AlN on the surface of Si to grow GaN can be used^32^. 1st step is low growth rate with high III-V ratio/pressure and 2nd is high growth rate with low III-V ratio/pressure to minimize the stress and dislocation defects. So, by controlling III-V ratio, pressure and temperature at which the GaN is grown on Si, the dislocation defects, and the stress at Si/GaN interface can be reduced to get high quality of GaN layer. The experiments in the past have shown significant results of obtaining 0.201 GPa biaxial stress as against the 0.397 GPa of biaxial stress^33^. Furthermore, there is one more method of melt back etching of Si during GaN growth. But this method will lead to high n-type doping background making it difficult to do p-type doping^34^. But since the proposed device does not need p-type doping, this type of technique can be suitably employed to achieve a quality GaN on Si. There are many other techniques which have been proposed to successfully integrate Si and GaN leading to successful fabrication of “GaN on Si” devices in research institutes like Indian Institute of Science (IISc) and Rochester Institute of Technology (RIT)^35–39^. The pocket region is made of Si_0.8_Ge_0.2_ having a bandgap of 0.997 eV. This is because, as Germanium concentration increases intrinsic carrier density increases and hence it would be better to choose a mole-fraction which can give high carrier density without reducing the mobility of carriers at the pocket-channel material junction for this device. Usage of Si_1-x_Ge_x_ mole fraction has also shown to reduce the gate to drain capacitance in the past which greatly effects gain bandwidth product (GBP)^40^.

The length of the device is taken as 60 nm with source/drain length of 15 nm and channel length of 30 nm with pocket occupying 2 nm of its length. The device dimensions and doping parameters used in the simulation are listed in Table 1. Several TCAD device models are used to make the device simulation closer to experimentation. Hydrodynamic model is used along with SRH model for 2D device simulation. PhuMob & Lombardi models are used for mobility; as the doping concentration is high, Fermi-Dirac model is used instead of Boltzmann characteristic model. Hurkx model is used for band-to-band tunneling (BTBT) analysis; effects such as scattering are neglected since the doping concentration is high. Non-Local model is used for the region interface of pocket and channel to consider tunneling in lateral direction. As the charges get trapped at oxide/Silicon interface during manufacturing process, material interface specific interface trap model is included with negative trap charge of -4.5×10^10^ Coulombs. The designed poc-DG-AJLTFET is simulated for varying gate voltage with drain voltage (V_ds_) of 1 V to obtain transfer characteristics, threshold voltage and its performance in subthreshold region.

**Table 1:** Parameter Spacing of the device.

Geometrical/doping parameter	poc-DG-AJLTFET
Gate Length (L_g_)	30 nm
Pocket Length (L_p_)	2 nm
Channel Thickness (T_ch_)	10 nm
Gate Oxide Material & Thickness (T_ox_)	HfO_2_-1 nm
Source, Drain, Channel & Pocket dopant-doping Concentration	Arsenic-1 × 10^19^/cm^3^
Source and Drain Material	GaN
Channel and Pocket Material	Si, Si_**1-x**_Ge_**x**_ (x = 0.8)
Gate-1, Gate-2 work-function (WF)	5.25 eV, 5 eV

**Figure 2:**
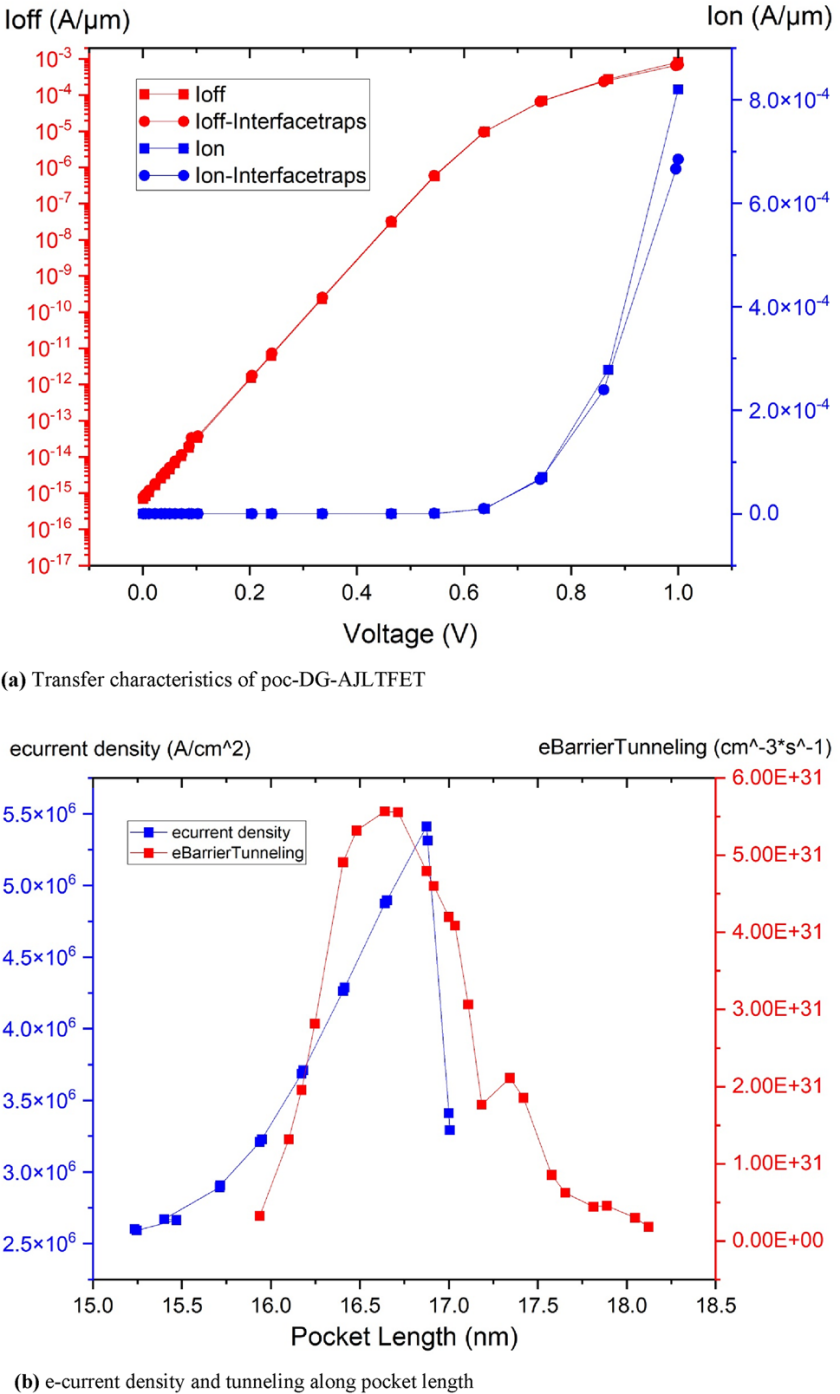
(**a**) Transfer characteristics of poc-DG-AJLTFET. (**b**) e-current density and tunneling along pocket length.

The transfer characteristics of the device with and without traps are shown in Figure 2a. From Fig. 2a it can be inferred that since the charges get trapped at polarity gate oxide and source interface, it limits the current from source to channel depending upon the type of trap charge. This degrades the electrical characteristics of the device in circuit level. So, unlike in conventional JLTFETs, the proposed poc-DG-AJLTFET does not have a polarity gate. But without polarity gate at source, the n^+^ doped source does not get converted into a p^+^ region, without which, there is no charge gradient in the source to channel regions as they both are doped with n^+^ dopants. This inhibits the charge carriers to mobilize from source to channel. So, both pocket and channel has been made p^+^ regions using different gate work functions for PG and CG respectively.

As the charge gradient is established in the device, it needs the right bandgap for the carriers to tunnel through. For this, the channel is made of Si and a low bandgap material, SiGe has been used as pocket to align the bandgaps of pocket-channel regions to facilitate the tunneling in the device. Also, the pocket under the influence of PG generates high current density near pocket-channel material junction and into the channel to improve I_ON_. As explained, both the tunneling and high electron current density along the length of the pocket can be observed from Fig. 2b.

Though I_ON_ is observed to be high, to improve I_ON_/I_OFF_, there are other factors such as effective mass (m*) of charge carriers that can be tuned^41,42^. I_ON_ of poc-DG-AJLTFET is based on BTBT mechanism and critically depends on the transmission probability T_WKB_ of the inter band tunneling barrier. Relation between T_WKB_ and drain-source current is given in Equation 1 which can be calculated using of Wentzel-Kramers-Brillouin (WKB) approximation and two band dispersion relation is given in Equation 2^43^.

1 $${\text{I}}_{{{\text{ds}}}} = { }\frac{{2{\text{q}}}}{{\text{h}}}{\text{ T}}\left( {\Delta {\text{E}}} \right){\text{F}}_{{{\text{integral}}}}$$

2 $${\text{T}}\left( {\Delta {\text{E}}} \right) \approx {\text{exp}}\left( { - \frac{{{\uppi }\sqrt {2{\text{m}}_{{\text{T}}} {\text{E}}_{{\text{g}}}^{3/2} } }}{{4{\text{q}}\hbar {\text{F}}\Delta }}} \right)$$

3 $${\text{SS}} = { }\left( {\frac{{{\text{dlog}}_{10} \left( {{\text{I}}_{{{\text{ds}}}} } \right)}}{{{\text{d}}\left( {\frac{{\Delta {\text{E}}}}{{\text{q}}}} \right)}}} \right)^{ - 1}$$

where $\Delta E$ is the allowed tunnel window contributing the tunneling of carriers, F_integral_ is the integral function of Fermi–Dirac distribution of source/drain, m_T_ is the effective carrier mass, E_g_ is the bandgap, q is the electron charge, $\hbar$ is the reduced planck’s constant, ${\text{F}}\Delta$ is the junction electric field and depends on λ which is the screening tunneling length and describes the spatial extent of the transition region at the source-channel interface shown in Eq. 4. So, by tuning the tunneling probability, better I_ON_ can be achieved^44–46^. With this conceptual observation, the 2 key factors, λ, m_T_ are essentially tuned in to get better results in terms of switching ratio.

4 $${\uplambda } = \sqrt {\frac{{{{\varvec{\upvarepsilon}}}_{{{\mathbf{Si}}}} }}{{{{\varvec{\upvarepsilon}}}_{{{\mathbf{ox}}}} }}} \cdot \sqrt {{\text{t}}_{{{\text{ox}}}} {\text{t}}_{{{\text{Si}}}} }$$

with gate1 work-function of 5.25 eV and gate2 work-function of 5 eV, the proposed Si_0.8_Ge_0.2_ JLTFET has better I_ON_/I_OFF_ current ratio as well as near to the ideal subthreshold slope and a very low drain-induced barrier lowering (DIBL) even with small oxide thickness of 1 nm. Since the work functions are different for the two gates, simulating both gates with same voltage brings better control of channel for this device. However, usage of independent gate configuration for better leakage control is being considered for future work.

The calibrated JLTFET TCAD models serve as an approximation of full-band atomistic calculation of JLTFET band-to-band tunneling current to generate the DC characteristics. The obtained DC characteristics (I_d_-V_g_) and AC characteristics (Capacitance behavior) from simulations are employed for generating circuit level symbols of the poc-DG-AJLTFET based on the Penn State Verilog-A model^47^.

**Figure 3:**
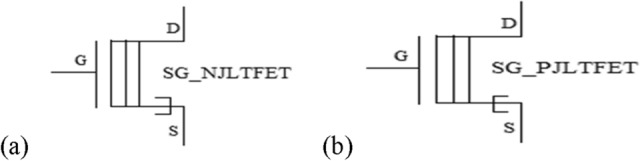
Symbol of shorted gate (**a**) SG_NJLTFET and (**b**) SG_PJLTFET.

The circuit symbols of shorted gate n-type JLTFET (SG_NJLTFET) and shorted gate p-type JLTFET (SG_PJLTFET) are shown in Fig. 3a,b.

The double lines inside the transistor indicate that the double gates are shorted by applying same gate voltage.

## Results and discussion

This section provides the implementation and analysis of QPSK system using poc-DG-AJLTFET. This includes two sub-systems named as pseudo-random binary sequence (PRBS) generator which uses linear feedback shift register (LFSR) principle to generate random bits in sequence for the first subsystem. This is then split by the second subsystem called as bit splitter circuits which outputs odd and even sequences based on the position of bits in PRBS output. So, as PRBS generator generates random sequence of binary bits using DFFs in linear feedback shift register configuration, an X-OR gate acts as feedback element.

The implementation of this system is done in 3 stages.

1. Realization and simulation of leaf cells which are inverter cell, NAND, and X-OR gates using n-type and p-type poc-DG-AJLTFETs.
2. Realization and simulation of DFFs using 4 NAND gates and an inverter cell.
3. Realization and simulation of PRBS generator and bit splitter using DFFs.

As each simulation is carried out, circuit performance metrics such as power dissipation, power consumption and delay are extracted using Cadence tool.

### Realization of leaf cells using poc-DG-AJLTFET

In this section, leaf cells mentioned in stage 1 are realized and simulated which are then used to construct DFFs.

#### Realization of inverter logic circuit

**Figure 4:**
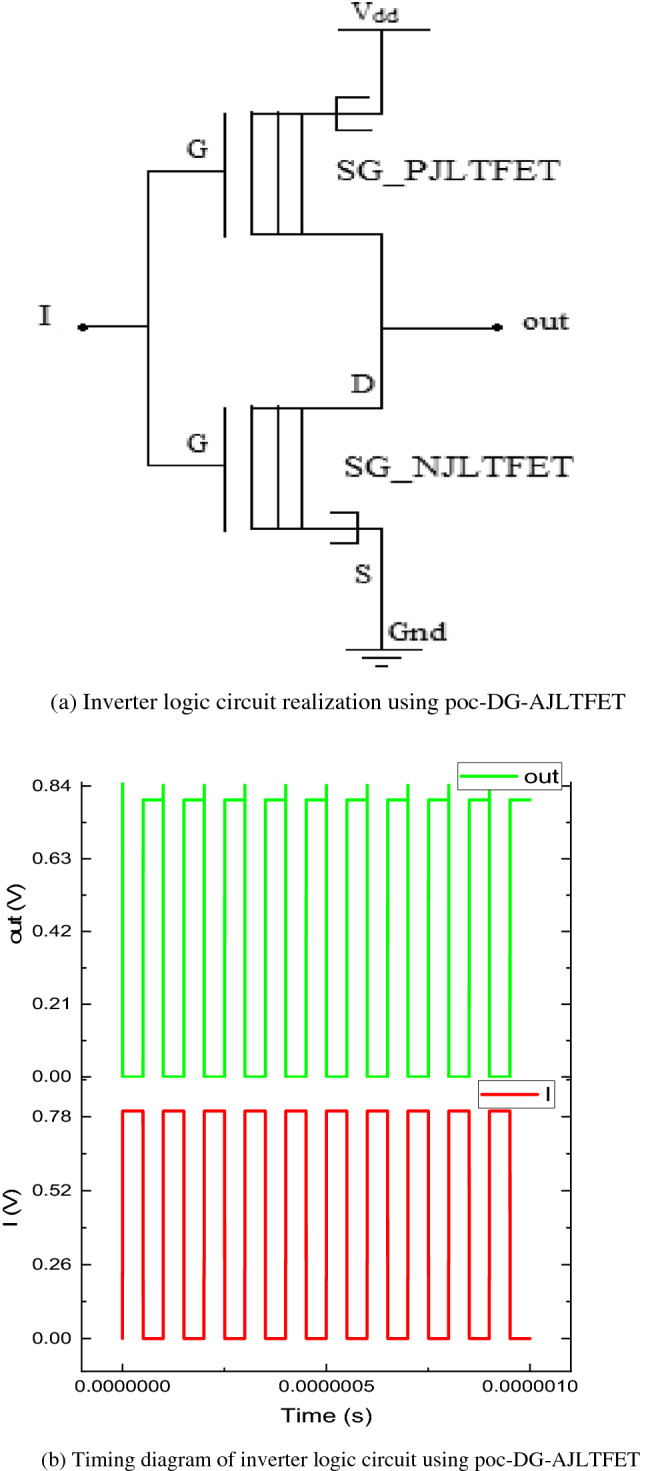
(**a**) Inverter logic circuit realization using poc-DG-AJLTFET. (**b**) Timing diagram of inverter logic circuit using poc-DG-AJLTFET.

The poc-DG-AJLTFET based inverter logic circuit is shown in the Fig. 4a. The inverter cell is made of a SG_PJLTFET in pull up network and a SG_NJLTFET in pull down network as in any MOS inverter configuration^48^. When input signal is logic high, SG_PJLTFET gets turned off and the path between supply and output is closed. This makes output logic low and when logic low input is given, SG_PJLTFET gets turned on making a path between supply and output which makes output logic high. The timing diagram of the inverter logic circuit is shown in Fig. 4b.

#### Realization of NAND logic circuit

**Figure 5:**
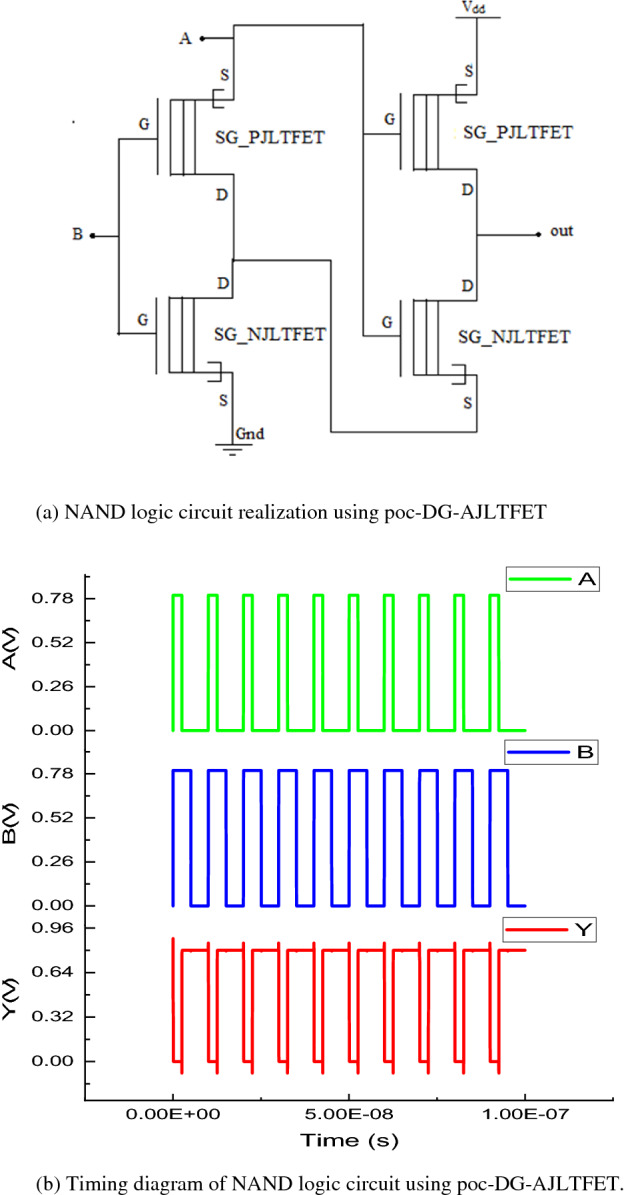
(**a**) NAND logic circuit realization using poc-DG-AJLTFET. (**b**) Timing diagram of NAND logic circuit using poc-DG-AJLTFET.

The poc-DG-AJLTFET based NAND logic circuit is shown in the Fig. 5a. The NAND gate has been constructed in such a way that there are minimum paths between the supply (V_DD_) and ground (Gnd) for different combinations of input. As part of this construction, signal A has been used as supply/input. When both input signals are logic low or when either of inputs is logic low, both SG_PJLTFETs get turned on and conducts a path between supply and output thereby, resulting in logic high output. In this case, there is only one path between supply output which reduces active power consumption as well. When both inputs are given logic high, both SG_NJLTFETs get turned on, resulting in logic low output. The circuit in both on and off states conserves power and so this type of configuration can be used in realizing low power cells. The timing diagram of NAND logic circuit is shown in Fig 5b.

#### Realization of X-OR logic circuit

**Figure 6:**
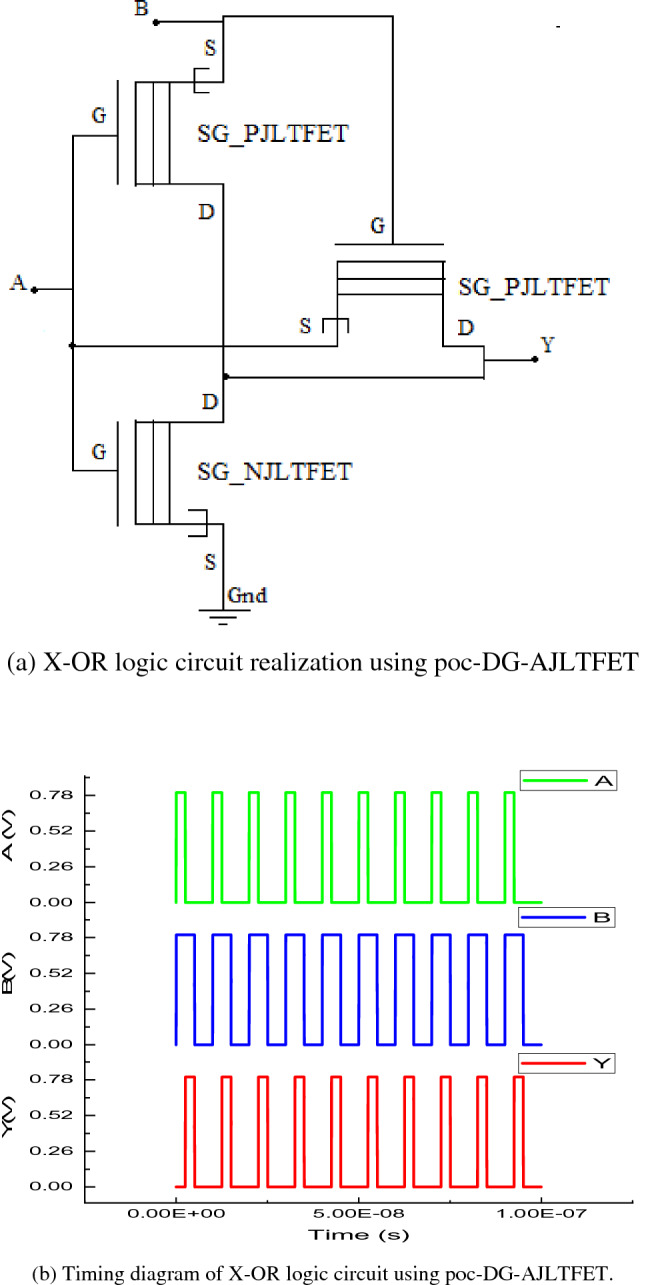
(**a**) X-OR logic circuit realization using poc-DG-AJLTFET. (**b**) Timing diagram of X-OR logic circuit using poc-DG-AJLTFET.

The realization X-OR logic circuit using poc-DG-AJLTFET is shown in the Fig. 6a. Here the X_OR logic circuit uses pass-transistor-logic (PTL) which is used to reduce complexity of the circuit^49^. With only three transistors, the X-OR logic circuit realized does not require supply voltage as well as complementary inputs (if complementary form of input is to be used, an inverter will again be needed for realization which increases complexity). When input B is given logic high, transistor SG_PJLTFET at the output side gets turned off, and the entire circuit operates like an inverter and complement of logic input A gets passed to the output. But when inputs A & B are given logic low, the output will be at V_TP_ instead of complete logic low. Similarly, when input A is logic high and input B is logic low, the output will be at V_DD_-V_TP_ instead of complete logic high. It can be noted that since this X-OR circuit is not used in cascade mode in realizing QPSK system, this did not cause any problem in simulations. The timing diagram of X-OR logic circuit is shown in Fig 6b.

### Realization of D flip-flop

**Figure 7:**
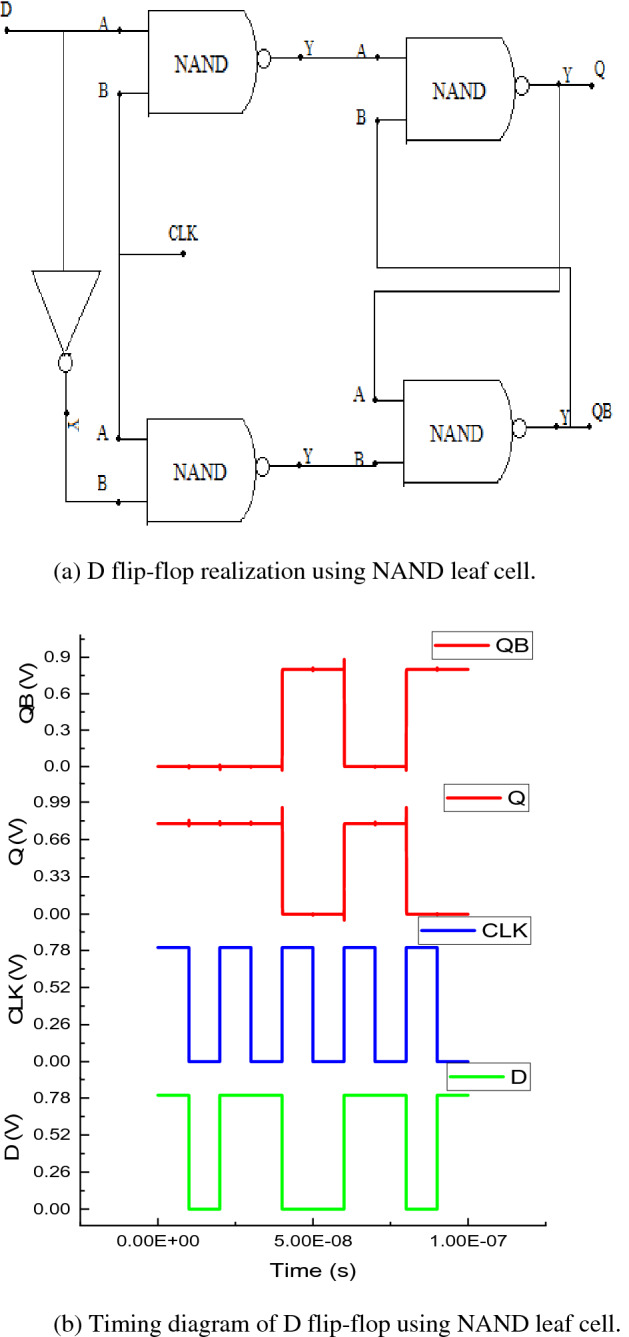
(**a**) D flip-flop realization using NAND leaf cell. (**b**) Timing diagram of D flip-flop using NAND leaf cell.

The poc-DG-AJLTFET NAND based D flip-flop is shown in Fig 7a. Assuming the initial logic states of outputs Q and QB to be logic low and logic high respectively, when the clock signal (CLK) and input signal D or the data signal is given logic high, the NAND gates leading to the path Q, will pass logic high to it and the NAND gates leading the path to QB will pass logic low. The timing diagram of DFF is shown in Fig 7b.

### Realization of QPSK system

This section provides implementation and simulation of QPSK system by connecting output of PRBS generator to input of bit splitter or even/odd sequence (ES/OS) generator.

#### Realization of PRBS generator

**Figure 8:**
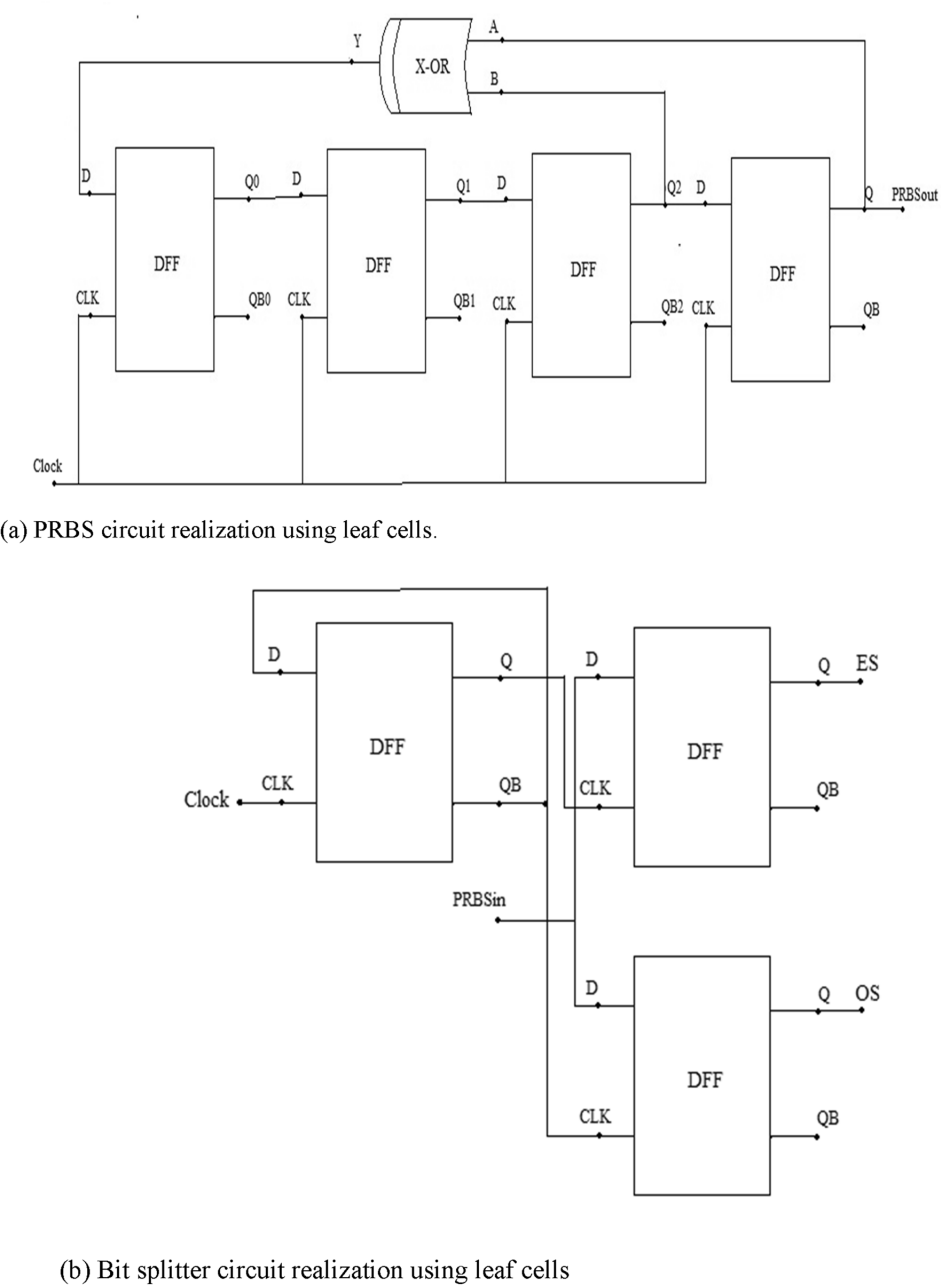

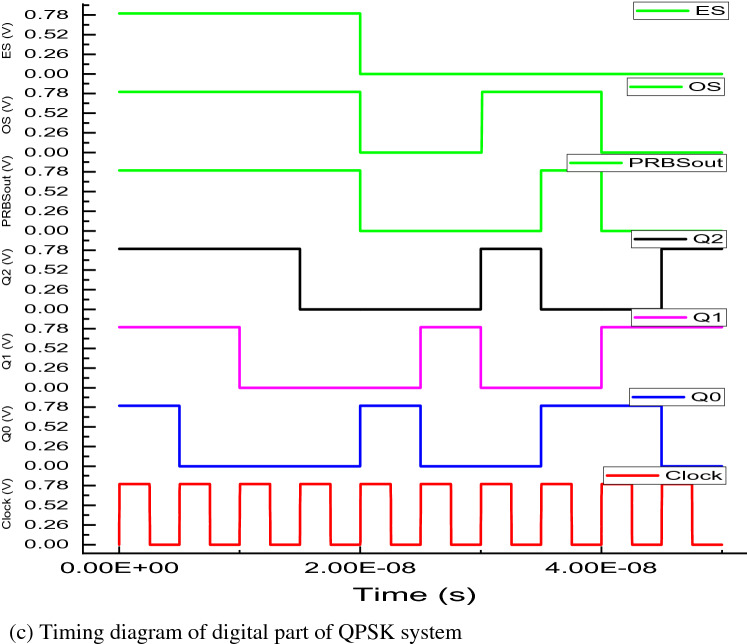
(**a**) PRBS circuit realization using leaf cells. (**b**) Bit splitter circuit realization using leaf cells. (**c**) Timing diagram of digital part of QPSK system.

The PRBS generator is realized using the leaf cells and is shown in Fig. 8a. A PRBS bit stream can be generated by connecting the DFFs using a LFSR configuration^50,51^. So, as each bit traverses through each flop, a certain bit delay is introduced until the data reaches last flop. The third flop output and the PRBS output is given to X-OR gate which generates difference output. This output of X-OR gate is fed-back as input data to generate random binary sequence. As 4 DFFs are used in this circuit, 15-bit sequence can be generated each time. If the sequence is 1 1 1 1 0 0 0 1 0 0 1 1 0 1 0, X-OR gate will generate logic high output, and this generates a new sequence of bits as 1 1 1 1 1 0 0 0 1 0 0 1 1 0 1. Each sequence corresponds to unique information in a communication system though there’s only 1-bit change at most significant bit (MSB) side.

#### Realization of ES/OS generator or bit splitter circuit

The bit splitter circuit has been constructed using the leaf cells and is shown in the Fig 8b. In this circuit, instead of giving a single clock signal to the flip-flops generating odd and even bits of data, a D flip-flop has been used as clock generator to eliminate the ill-effects of clock-skewing and unnecessary timing violations. The first D flip-flop (Clock generator) outputs Q and QB which are then connected to odd/even sequence generating flip-flops so that both flip-flops generate the bits of data in alternating cycles of clock using the data coming in from the PRBS circuit. The then generated odd and even bits of data are used to generate the QPSK modulated signal. The timing diagram of bit splitter circuit is shown in the Fig 8c.

## Performance analysis of QPSK system

This section provides the overall performance analysis of QPSK system. The designed poc-DG-AJLTFET is analyzed to check whether the device is providing necessary performance metrics that are required for a communication system. In device level, the key DC parameters are I_ON_, I_OFF_, and SS. As these metrics are direct contributors to the device performance, they are optimized by choosing oxide thickness as low as 1 nm which effects λ and by tuning both of the gate work functions; SS is observed as per the Equations 3 & 4 which is given in Table 2. As per Equation 2, electron tunneling mass is kept at 0.1 times of rest mass (m_o_) and hole tunneling mass is kept same as rest mass at m_0_. This ensures high tunneling of carriers in between bands. Also, the active layer/channel width has been increased to 10 nm to increase the ON current. With this, a SS of ~60mV/decade and switching ratio of 1.218×10^12^ has been observed. Also, because of low C_gd_, the trans-conductance g_m_ obtained is high which automatically resulted in high GBP shown in Table 2. Drain induced barrier lowering (DIBL) and GBP are calculated using Equations 5 & 6 respectively. The DIBL of the device is approximately close to zero (2.75 mV/V) due to high doping concentrations and also due to the absence of junctions. Equation 6 is used to calculate DIBL at drain voltages of 1 V and 0.2 V. It is to be noted that even with thin oxide, the leakage current is very low, and that the switching ratio is very high.

5 $${\text{DIBL}} = { }\frac{{{\text{V}}_{{{\text{t}}2}} - {\text{V}}_{{{\text{t}}1}} }}{{{\text{V}}_{{{\text{D}}2}} - {\text{V}}_{{{\text{d}}1}} }}$$

6 $${\text{GBP}} = { }\frac{{{\text{g}}_{{\text{m}}} }}{{20{\uppi } \times {\text{C}}_{{{\text{gd}}}} }}$$

**Table 2:** Device level performance metrics.

Device Parameter	Value
V_t_	0.4675 V
I_ON_	1.643 mA
I_OFF_	1.348 fA
I_ON_**/**I_OFF_	1.218 × 10^12^
SS	60.3 mV/decade
DIBL	2.75 mV/V
C_**gd**_	68.87 aF
g_**d**_	11.82 mS
g_**m**_	13.62 mS
R_**out**_	7.39 KΩ
Intrinsic gain (g_**m**_ × R_**out**_)	100.65
f_**t**_	803.82 GHz
GBP	3.147 THz

Previous works by Goswami et al and Aghandeh et al, could not produce high I_ON_/I_OFF_, high f_t_, and a high g_m_ all at the same time. It is worth mentioning that, to obtain better results in all performance parameters, the a-symmetricity of the device with GaN material and gate oxide of HfO_2_/1 nm thickness have helped to achieve a high f_t_ of ~ 800 GHz. As the device is asymmetric, it is able to achieve reduction of leakage by order of 10^3^ and increase I_ON_ at the same time by order of 10^4^ compared to conventional symmetric DGJLTFETs. Table 3 provides an insight of performance achieved by the proposed device as against previous devices^52,53^.

**Table 3:** Performance comparison of proposed device with the state-of-the-art DG-JLTFETs.

Device parameter	References			
	Sharma et al. ^26^	Goswami et al. ^52^	Aghandeh et al. ^53^	This work
Oxide/t_ox_	SiO_2_/2 nm	HfO_2_/2 nm	SiO_2_/2 nm	HfO_2_/1 nm
I_ON_/I_OFF_	~ 10^9^	~ 10^13^	~ 10^8^	~ 10^12^
f_t_ (GHz)	2.9	7	12	800
g_m_	~ 3.8 µS	~ 1 mS	~ 3 mS	13.62 mS

### Circuit level performance metrics

The performance metrics being considered in the circuit level are power consumption, dissipation and propagation delay and the procedures to calculate these metrics are provided in Cadence manual^54^. For propagation delay, the key factors are rise and fall times of a circuit. As shown in Equations 7 and 8, rise and fall times depend on factors such as C_ox_, g_m_, and V_TH_. Since a QPSK system can also have large load capacitances, rise, and fall times can be very high leading to high delay in communication. To avoid that, it is necessary to have lower V_TH_ and high g_m_, which are already achieved and can be seen in Table 2.

7 $${\text{T}}_{{{\text{PLH}}}} = { }\frac{{{\text{C}}_{{\text{L}}} }}{{{\text{g}}_{{m_{{\text{p}}} }} { }\left( {{\text{V}}_{{{\text{gs}}}} - {\text{V}}_{{{\text{TH}}}} } \right)}}$$

8 $${\text{T}}_{{{\text{PHL}}}} = { }\frac{{{\text{C}}_{{\text{L}}} }}{{{\text{g}}_{{{\text{m}}_{{\text{n}}} }} { }\left( {{\text{V}}_{{{\text{gs}}}} - {\text{V}}_{{{\text{TH}}}} } \right){ }}}$$

where C_L_ is the load capacitance, T_PLH_ and T_PHL_ are rise and fall times respectively. The relation between rise, fall times and propagation delay can be observed from Equation 9.

9 $${\text{t}}_{{{\text{pd}}}} = { }\frac{{{\text{t}}_{{{\text{PHL}}}} + {\text{ t}}_{{{\text{PLH}}}} }}{{2{ }}}$$

In the power metrics, two powers are mentioned and analyzed. One is the dynamic or active power consumption which is the power consumed when the inputs are given to the circuit, and another is power dissipation which is wasted power as the energy slowly dissipates either through load capacitance or internal gate-drain capacitance when there is no input. These power metrics are analyzed using Equations 10 and 11. The active power consumption is directly dependent on the supply voltage and frequency of operation. Power consumption and power dissipation are indicated with P_C_ and P_D_ respectively with input frequency (f_i_).

10 $${\text{P}}_{{\text{C}}} = \left( {{\Sigma }\left( {{\text{leakage}}\;{\text{current}}} \right) \times {\text{ V}}_{{{\text{DD}}}} { }} \right) + {\text{ C}}_{{\text{L}}} { } \times {\text{ V}}_{{{\text{DD}}}}^{2} { } \times {\text{ f}}_{{\text{i}}}$$

11 $${\text{P}}_{{\text{D}}} = {\text{ C}}_{{{\text{pd}}}} \times {\text{V}}_{{{\text{DD}}}}^{2} { } \times {\text{ f}}_{{\text{i}}}$$

12 $${\text{C}}_{{{\text{pd}}}} = \frac{{{\text{IDS}}}}{{{\text{V}}_{{{\text{DD}}}} \times {\text{f}}_{{\text{i}}} }} - {\text{C}}_{{{\text{Leff}}}}$$

13 $${\text{C}}_{{{\text{L}}_{{{\text{eff}}}} }} = {\text{ C}}_{{\text{L}}} { } \times {\text{ N}}_{{{\text{SW}}}} { } \times { }\frac{{{\text{f}}_{{\text{o}}} }}{{{\text{f}}_{{\text{i}}} }}{ }$$

As the leakage current is less in sub-threshold region, leakage current can be neglected in Equation 10. From Eqn. 11, as V_DD_ and f_i_ are fixed, C_pd_ is a factor which can be altered to produce low dissipation by having as high gate-drain capacitance as possible which can be seen from Equations 12 & 13. But designing a system to have high load/ gate-drain capacitance can lead to poor fan-out capability. So, the obtained 68.87 aF is optimal and helps in improving the fan-out of the system. This gives an advantage to propagate the output signal to multiple loads. So, this is a trade-off between fan-out and power dissipation for the designed QPSK system. Though there is a trade-off between fan-out and C_gd_, the power dissipation for the QPSK system is still in the range of femto watt. Table 4 gives the performance metrics in circuit level.

**Table 4:** Circuit level performance metrics.

Logic gates	Propagation delay	Power consumption (pW)	Power dissipation
Inverter	8.70 ps	11.54	5.16 aW
NAND gate	7.63 ps	14.03	8.72 aW
X-OR gate	1.07 ps	9.38	1.37 aW
D Flip-Flop	9.14 ps	23.44	17.41 aW
PRBS generator	46.72 ps	67.32	68.62 fW
Odd/Even Stream generator	ES:58.21 ps OS:76.38 ps	43.25	85.43 fW

From Tables 2 and 4, it can be said that with the designed poc-DG-AJLTFET in QPSK communication system, consumption of power is 168.96 pW which is very much low as against the literature of 220 mW & 1.3 mW^55,56^. For X-OR gate, the logic transitions are high since it is a key element in generating continuous random sequences from PRBS circuit. So, for X-OR gate PTL has been applied which contributed to reduce the power consumption. It can be observed that with the usage of multiple techniques for low power in both device and circuit levels, overall QPSK communication system can be said to have produced enhanced performance for satellite communications.

## Conclusion

In all real-world devices, interface traps deteriorate the performance of the device. Therefore, in this work, a new poc-DG-AJLTFET has been designed to have negligible effects from interface traps and to perform better in both analog and digital circuit applications. Compared to conventional JLTFET devices, this structure eliminates the need for polarity gate and uses pocket region as both facilitator of tunneling and in reducing the tunneling barrier. So, this work offers a futuristic device with less variations of material usage to provide higher order of feasibility for manufacturing. The designed device is then optimized to achieve high I_ON_/I_OFF_ ratio and an ideal SS. With high I_ON_/I_OFF_ ratio in the order of 10^12^, high trans-conductance to drain current ratio of 8.28 S/A is achieved. As the device has high GBP of 3.147 THz, the device can produce a stable intrinsic gain of ~100 over wide range of frequencies with data rates up to 0.2 Gbps. The QPSK system is then implemented with this device which consumes only 55.28 pW of average power consumption with ability of propagating the output in a maximum of 123.1 ps, numerically a significant improvement in terms of power consumption in the order of 10^8^-10^9^ times has been observed. This greatly pushes the implemented system to be chosen for satellite communication systems that use QPSK method to transmit data or information. In all conventional QPSK systems, there is always a trade-off between power consumption and spectral efficiency. But now, the trade-off has been clearly eliminated and both the proposed device and implemented system achieved great results in all aspects of performance. Thus, it can be concluded that the proposed device and implemented system can produce significant performance wherever ultra-low power is preferred with high bandwidth and low latency.

## Data Availability

All data generated or analyzed during this study are included in this published article. Any other required data if requested by the reviewers shall be added to the manuscript.
